# Food neophilics’ choice of an ethnic restaurant: The moderating role of authenticity

**DOI:** 10.1371/journal.pone.0281453

**Published:** 2023-05-18

**Authors:** Inda Premordia, Timea Gál

**Affiliations:** 1 Doctoral School of Management and Business, Faculty of Economics and Business, University of Debrecen, Debrecen, Hungary; 2 Institute of Marketing and Commerce, Faculty of Economics and Business, University of Debrecen, Debrecen, Hungary; Shenzhen University, CHINA

## Abstract

This study attempts to identify the salient factors affected by food neophilia and its interaction with demand authenticity in the choice of an ethnic restaurant. By undertaking a series of multivariate and univariate analyses between these two predictors and five key dining attributes: food quality, service quality, FLEs attitude, atmosphere, and price, it is revealed that restaurant customers consider different factors in their buying decision process, depending on individuals’ food neophilia level, needs for authenticity, and demographic characteristics. The results show that authentic quality of food, authentic atmosphere, friendly and prompt service encounters are the most important factors. The findings further suggest that price sensitivity is higher in the market with a low to moderate need for authenticity. Cultural backgrounds, on the other hand, seem to influence how customers embrace roles and professional skills of the frontline staff rather than customer-employee relationship. Given the lack of research in examining food neophilia in the ethnic restaurant selection empirically, this study allows a better understanding of this market segment which contributes to the body of knowledge in the field of food consumption and preferences as well as offers useful insights for ethnic restaurant businesses.

## Introduction

Dining out preferences vary throughout different demographic and psychographic segments. It is not limited only to daily food choices, but also restaurant selection criteria which stretches from menu types, service styles, to price ranges. Some individuals are more open to enjoy diverse flavours of cuisines, while some others are more reluctant to consume a broader variety of food. With respect to dining place selection, the salience of restaurant dimensions such as food quality, service quality, ambience and price may differ considerably between the customers. To develop an ideal service model in the ethnic foodservice industry that meets and exceeds customer expectations, information on the importance of different restaurant attributes is—therefore, essential.

The past decade has seen a major shift in food preferences and the practice of eating. Everyone now is dining out for several motives: sociocultural-utilitarian-hedonic. What people eat is no longer relevant only in a utilitarian context, but on a larger scale, it is also a bridge that eliminates the barriers to get closer to and provide an insight of a culture. Food consumption has become an eminent symbol of one’s identity [1] as well as cultural identity [2].

Food as a symbol that represents cultural identity cannot be separated from traditional food, family food traditions or ethnic food. Ethnic food refers to the nuances of food in relation to attitudes, behaviours, values and beliefs of a distant culture that are a manifestation of its cultural traditions or heritage, religion or national origin [3]. Outside of its respective ethnic groups or dominant cultures within which a particular tradition exists, ethnic food is considered as novel. While ethnic food market was once previously regarded a niche market to a few selected consumers, nowadays is seen as a global emerging market that serves for the need of not only its communities and co-ethnics, but also the taste buds of the host country’s native as well as immigrants of different national backgrounds [4].

[5] explained an increasing trend towards ethnic food for various consumer segments across sociodemographic characteristics. For people within these segments, ethnic food expresses their cultural interest which has the ability to evoke certain emotions and a feeling of belongingness towards different cultures, including exotic places, ingredients, flavours, textures, aromas, and sensation. For others, ethnic food is a reflection of being adventurous, audacious and unconventional.

The spread of ethnic food around the world is capacitated through different factors, such as migration and tourism exchange among other things. As food traditions and heritage are generally passed on through generations, immigrants bring their foodways with them when they leave their homelands and sticking with the convention is a way of preserving their culture around food. This in turn induces culturally diverse societies which also offer business opportunities to both local and global marketers. Globalization of commerce and the continual rise in intercultural interactions have contributed to an expansion of our palates and culinary awareness as well as an increasing demands in terms of food variety. As such, cuisines from different cultures become more accessible and the vast number of ethnic restaurants can be found easily in comparison to marketplaces in a monocultural environment. In other words, an increase in international travel; inbound-outbound tourism; and immigration have encouraged “the hybridization of food models” [6].

In predominantly multicultural marketplaces (i.e., America, Canada, Australia, UK and European countries), the ethnic subcultures experience cultural assimilation through a multifarious form of application. Subsequently, there is a rapidly growing demand for authenticity. Generally, the role of authentic quality is an important element of customer evaluation of ethnic food and ethnic restaurants. In extant research on ethnic restaurants, researchers have investigated the significant positive impacts of authentic experiences on customer attitudinal and behavioural outcomes [7, 8]. [9] also pointed out that a lack of authenticity is one of the determinants that contributes to business failure in the service and hospitality industry. When customers determine whether ethnic food or ethnic restaurants are authentic, their evaluation depends on perceived authenticity with the reference from the image formation of their past experiences [10]. According to [7, 11], customer perception of authenticity is developed based on the uniqueness level of a product or service attribute in their assessments. [7], further noted that uniqueness is often associated with ‘authenticity’.

Defining the most salient restaurant attributes has been a topic of sustained activity in this domain’s literature, but there is little consensus on what may work best for the food neophilics’ dining attributes in choosing ethnic restaurants, specifically in Hungary. As it holds an increasing population of foreigners from other European countries, Africa, America and Asia who live in Hungary for business, diplomatic and educational purposes as well as for family reasons, it is crucial for ethnic restaurateurs in Hungary to understand customers’ dining out preferences. Accordingly, this study intends to narrow down theoretical and practical gaps by developing a model with a special focus on food neophilia. In detail, the current study investigated:

**RQ1 What are the most salient dining attributes affected by food neophilia in the choice of an ethnic restaurant?**
**RQ2 Whether demand authenticity moderates the causal relationship between food neophilia and key dining attributes?**
**RQ3 Whether demographic characteristics also affect the relative importance of the key dining attributes?**

The results of this study contribute to the previous literature by advancing knowledge on which dining attributes influence food neophilic individuals to choose an ethnic restaurant specifically, as well as examine whether authenticity plays a role in the relationship between openness level to try novel food and generally accepted dining attributes. This study also contributes to the field by providing practical information that will enable restaurateurs to be better prepared to cater for this market segment.

## Literature review

### Food neophilics: ‘The adventurous’

Different interconnected factors contribute to describe buying and consumption decisions, particularly regarding exotic foods. These factors can be either contextual, such as cultural or social; correlated with individual sensation-perception-cognition, such as fear of aversive consequences of eating a certain food or finding a particular type of food disgusting; or related to individual traits. The openness to experience ethnic foods seems to be determined by an individual trait known as food neophilia, which is defined as an acceptance or adventurousness to eat novel foods, a construct that can be considered to be the converse of neophobia. The measurement of these traits is served as an important indicator to analyse the willingness to accept the elements of novelty in a highly diverse multicultural society.

Neophilic individuals, the love of novelty and the exploration of the new—enjoy a wide variety of foods, the taste buds that crave for a great range of different foods [12, 13]. In a qualitative study by [14] it is found that people with neophilic tendencies generally associate experiencing novel foods with pleasure, diversity, and sophistication. Many separate themselves from the majority of people and view experimental eating as a way of refining their lives.

Furthermore, food neophilia may also be linked to acquiring a different appetite physiology, which enables individuals to explore food with more excitement [15] as experiencing novel foods can satisfy neophilic individuals [16]. Some neophilic individuals seem to take irrational, euphoric and bold pleasure in overstepping the borderline between “edible” and “nonedible”. Many individuals even highlight that they regularly try to order the most unusual items on restaurant’s menus when eating out [14]. This can be explained by what [17] stated in their study that “restaurants sell meanings as well as food.” As such, restaurants provide safe environment and right set of circumstances for people to consume unfamiliar food. [18] expressed that restaurants allow people to pursue “the new, exciting, and interesting through these mediated, circumscribed gestures that allow us to contain the challenge of the exotic while simultaneously indulging in it.”

Based on the theory of cultural distance [19], allocentric tourists, those who are constantly seeking for new experiences, have a special interest in tasting the local cuisine of culturally distant markets or destinations, affected by a high degree of food neophilia [20]. Additionally, [21] suggested that the growing of new cuisines and the globalization of local cuisines in many places around the world are particularly influenced by individuals with higher novelty-seeking tendencies as a travel motivator considering that food neophiles seem to be more inclined toward new food adventures [22]. Many of the neophilic individuals consider savouring local dishes from destinations they visit during their journey to be a crucial part of traveling. Eating indigenous food of different places is regarded as part of an authentic tourism experience and an approach to learn more about different cultures. Further [6] explained that the acceptance of and willingness to try new foods seem to be influenced by the openness to other cultures. In relation to ethnic restaurants, [23] research argued a growing demand for ethnic restaurants and ethnic food markets in the US. It is revealed that cultural familiarity and food-related personality traits influence consumer use of ethnic food. Additionally, [4] also found that many US consumers accept novel ethnic foods due to the influence of ethnic diversity, globally sourced food, cultural experiences, and exposure through media. In a cross-national study on Korean and US consumers, [24] indicated that Korean consumers exhibit higher level of food neophobia than US consumers. In a study that examines tourist food consumption in Hong Kong by [16], more than one third of the participants express the desire to seek food and dining experiences to obtain local cultural knowledge.

### Generally accepted restaurant dining attributes

[25] defined perceived service quality as the gap between customers’ pre-conceived expectations and their evaluation on the overall service excellence. When choosing a restaurant, there is a wide array of service attributes that customers take into consideration in their judgment, such as food quality, service quality, atmosphere and price. Several researchers have elucidated that it is essential that restaurateurs recognize the relative importance of each restaurant dining attribute as customers make decisions based on the degree of importance attached to each attribute [26–28]. An extant literature has identified different restaurant dining attributes that are of vital importance to customers when choosing a restaurant. For example, [29] analysed food taste, serving portion, nutritional content, service quality, variety of menu, atmosphere, price, cleanliness, and hygiene. [30] found that customers perceive restaurant dining attributes differently in terms of food quality, service quality, type of cuisine, atmosphere and price. In more recent studies, [31] indicated the importance of the following restaurant dining attributes: food quality, food safety, variety of menu, cleanliness, service quality, timeliness, frontline staff attitude, perceived value for money, price, and location. [32] explained six most important factors that contribute to customer satisfaction: (1) food quality, (2) service quality, (3) ambience, (4) price, (5) menu, (6) design & décor, listed in order of their importance. [33] focused on the following influential factors when customers choosing a restaurant: food quality, food safety, food variety, service quality, timeliness, value for money, atmosphere, convenience, security, and additional facility. [34] examined variety-in-menu, gourmet taste, upscale image, hospitality, price, design & décor, ambience, responsiveness, and food presentation.

Because resources are limited, yet the numbers of restaurant choice factors demanded by customers are indefinite, a shared understanding of the relative importance of restaurant choice factors can provide guidelines for restaurateurs in enhancing their offering [35]. Thus, restaurant managers can prioritize and allocate their resources based on the most important attributes that affect the customers’ selection of restaurants [36]. Research in the service marketing field has reported three generally accepted restaurant dining attributes that have a significant impact on customer satisfaction towards restaurant dining experience: food quality, service quality, and atmosphere [37, 38]. Food quality refers to food-related aspects including food presentation, taste, variety of menu, fresh ingredients, and availability of healthy options [39]. Food quality is a crucial determinant of customer satisfaction and in post-dining behavioural intentions, such as word-of-mouth and re-patronage [28, 40–43]. Equally, service quality can significantly contribute to customer satisfaction and is found to be a strong factor in affecting customer loyalty, which is fundamental to business success [15, 44]. As customer buying decision process is not based solely on a single attribute, but on their evaluation towards the overall experience of a service or product, service quality can comprise of both tangible and intangible dimensions. According to DINESERV and SERVQUAL models, tangible dimensions consist of the appearance of physical facilities, personnel, equipment, and communication materials [45], while intangible dimensions include how the service is delivered, such as reliability and assurance (timeliness, no order errors, consistency) as well as empathy and responsiveness which related specifically to the frontline staff attitude (friendliness, politeness, willingness to help). [40] found that the service quality attributes for a consistent service as well as friendly and helpful employees significantly affect customer satisfaction and are the most important attributes influencing future behavioural intentions.

One tangible attribute of restaurant dining experience, atmosphere, has also been reported to be a powerful factor in customer satisfaction. According to [46], atmosphere creates customer perception of surrounding space, and the customer perceived value of the surrounding space shapes their affective states, which may alter or influence their buying behaviour. Researchers have long studied the impact of atmosphere on customer emotions, attitudes, and behaviours. [47] examined a model that establishes the relationship of physical environment and customer behaviour and emphasized the importance of the key components of retail atmosphere on customer buying decisions [48, 49]. The retail atmosphere attributes of interior design, decor and cleanliness were found to be the most critical in affecting behavioural intentions. [50] observed that customer satisfaction with the dining experience is also dependent on the convenience of access and parking availability.

In addition to the quality attributes of restaurants, perceived price fairness or value for money has a strong impact on customer satisfaction and behavioural intentions. How customers perceive product quality and monetary sacrifice stems from the customer perception of price. Customers believe that the higher the price, the better the quality as price acts as a signal of a service or product quality. In terms of restaurant selection, customers are often willing to pay premium prices for the high-quality, unique, and memorable dining experience as in the case of a fine dining restaurant. Research suggests that customer perception of the highest and the lowest price for a service or product impacts their buying decision. In their study on ethnic restaurants, [40] found that price fairness positively impacts customer satisfaction and loyalty, where the perceived price unfairness may lead to negative attitudinal and behavioural responses, such as dissatisfaction, complaining, brand switching and negative WOM. A study by [15] of the dining experience attributes in a university dining facility revealed the importance of price factors in influencing customer satisfaction and post-dining behavioural intentions. Other existing studies have also measured price in relation to the authenticity of food and atmosphere [51, 52].

This study examines five generally accepted restaurant dining experience attributes: food quality, service quality (reliability, assurance, and responsiveness), frontline staff attitude (appearance, empathy, and friendliness), atmosphere and price as the outcome variables. Additionally, according to [53, 54], consumers’ restaurant selections may also be influenced by socio-demographic factors as age, gender, nationality, income and marital status. Table 1 summarizes existing literature review of the relative importance of dining attributes that particularly examine five generally accepted dining attributes.

**Table 1:** Key authors and key attributes of restaurant dining experiences.

Authors (Year)	Key attributes				
	Food quality	Service quality	FLEs attitude	Atmosphere	Price
[54]	√	√	√	√	√
[32]	√	√	*	√	√
[33]	√	√	*	√	√
[53]	√	√	√	√	√
[55]	√	√	√	√	√
[56]	√	√	*	√	√
[57]	√	√	√	√	√
[26]	√	√	√	√	-
[58]	√	√	√	√	√
[27]	√	√	*	√	√
[59]	√	√	√	√	√

*Note*:

*Combined with service quality as a single attribute.

### Ethnic restaurants and the role of authenticity

According to [60], ethnic restaurant is defined as one that prepares and serves food that is perceived as novel and originating from a culture or heritage of certain ethnic groups. [61] defined ethnic restaurant as a restaurant that offers cuisines of a specific place combined with an authentic diningscape which mainly consists of physical objects found in that respective place including atmosphere and design and décor. The main purpose for consumers to visit an ethnic restaurant is to indulge themselves with food from another culture without traveling to the place where it originated. Thus, ethnic restaurants provide consumers from different culinary traditions an opportunity to taste new flavours as well as to experience new cultures [8, 62]. Similarly, [7] noted that consumers who dine at an ethnic restaurant, in general, would expect to devour on unique food and cultures through the dining experience. Past studies showed that consumers eating at an ethnic restaurant seek for an authentic dining atmosphere and that the authenticity is of a high importance in generating consumer satisfaction [63, 64]. For ethnic restaurant managers, it is crucial to know which dining attributes are most salient for consumers in order to optimize priority-setting to those attributes. Extant research identified food taste and appearance [4], service quality [28, 39, 65], price and value for money ratio [28], and price and convenience [66] as the main attributes in influencing ethnic restaurant preferences. In addition, food attitude is also considered as a strong factor in individual preferences and is able to predict food-related behaviour [67–69]. Earlier study by [70] found that attitudes play a big role in consumer consumption and buying behaviour toward ethnic food. Consumer acceptance toward ethnic food have been improved due to the following factors: (1) growing numbers of ethnic restaurants [71, 72]; (2) perceived image of ethnic food, such as healthy, fresh, and authentic [71]; (3) desire to learn about different cultures [8]; and (4) growing interest in healthy diet [73].

When talking about ethnic food, it cannot be separated from its authenticity. Authenticity refers to the quality of being ‘valid’ due to its originality, exact recreation, or representation, or “containing an original stamp or seal of approval” [74]. In the field of hospitality and tourism, authenticity has been perceived as a universal value and a strong driving force that persuades individuals to travel to distant places as well as different time zones [75]. In general, the authenticity has been classified into three perspectives: objective, constructive, and postmodern. [76] explained that these three perspectives can be useful to understand the authenticity in the context of ethnic restaurants. According to objectivist’s logic, in relation to ethnic restaurants, food ingredients, food preparation processes, restaurant’s design and décor, staff’s physical appearance and personality can be regarded as authentic when these factors comply to the culture of origin. Constructivists, on the other hand, propose that authenticity can only be assessed subjectively [77]. [78] argued that demand authenticity level depends on how each consumer perceives and makes meaning of what they see and experience. Perceived authenticity is measured dependent on the context.

Postmodern consumers are likely to evaluate the authenticity of their experiences based on their emotional judgement. In relation to ethnic restaurants, customers will perceive a restaurant as authentic if the overall experience is aligned with their desired emotions. Existing research reports a highly significant influence of perceived authenticity on consumer behaviour [40, 79]. For example, [75] found that perceived authenticity of tourists has a strong effect on future intentions. Other scholars pointed out that the impact of perceived authenticity on loyalty is greater than satisfaction [80, 81]. Further, it is found that perceived authenticity toward both food and restaurant setting may increase customer satisfaction and re-patronage intention, specifically in ethnic restaurants [40, 79].

### Conceptual framework

**Fig 1:**
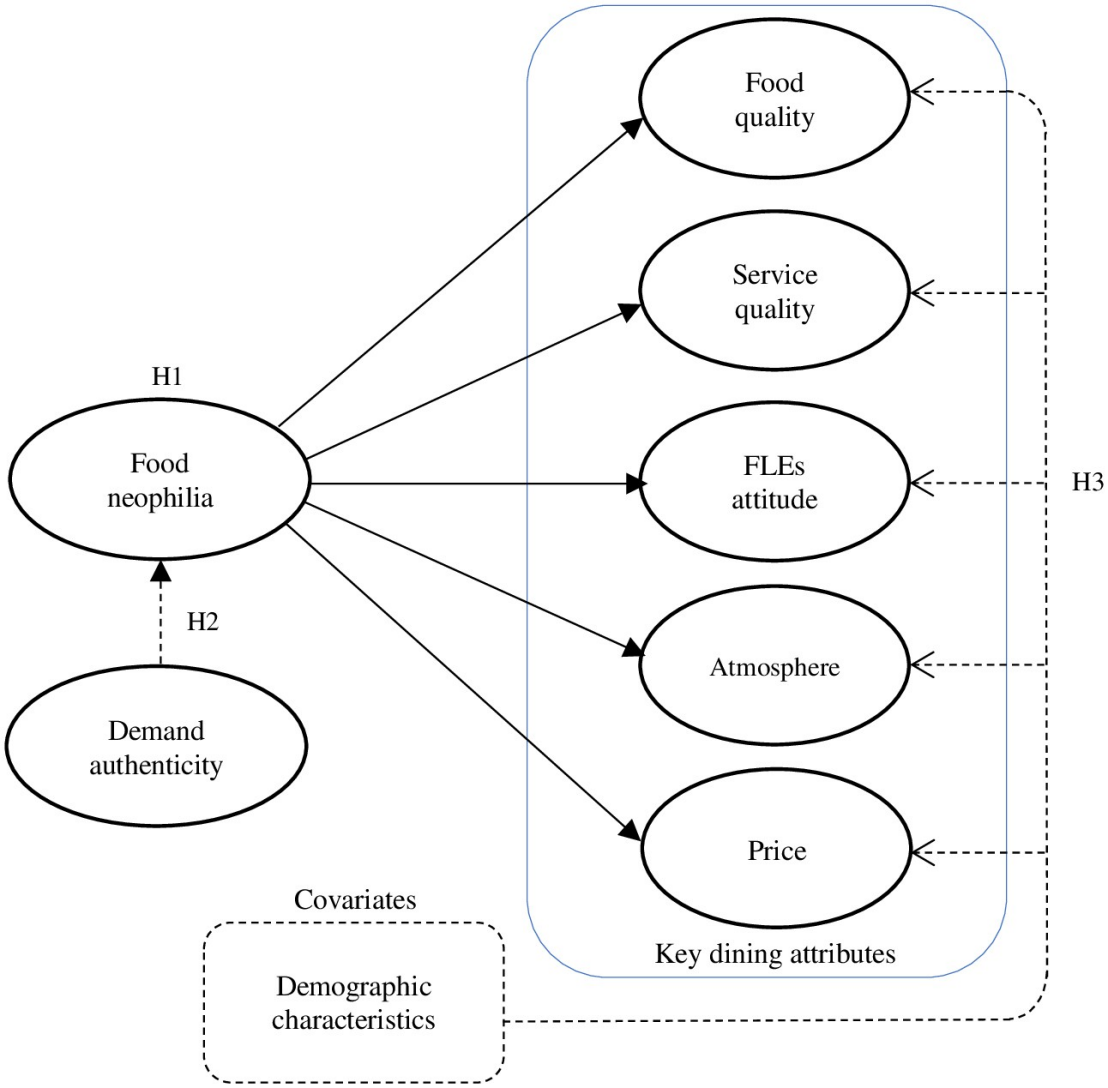
Conceptual framework.

Fig 1 highlights the diagram of conceptual framework used in this study. The framework is derived from the review of the research literature previously discussed. The framework consists of eight main constructs namely Food Neophilia and Demand Authenticity as independent variables; Food Quality, Service Quality, FLEs Attitude, Atmosphere and Price as dependent variables; and Demographic characteristics of the participants as covariates. The aim of this study is to determine the effect of food neophilia level as well as demand authenticity level on the relative importance of the five main restaurant dining attributes with a moderating effect of demographic profile of the participants.

Thus, based on the conceptual framework, following hypotheses were developed:

*H1 Food neophilia has a positive effect on the relative importance of*: *(a) food quality; (b) service quality; (c) FLEs attitude; (d) atmosphere; and (e) price in the choice of an ethnic restaurant*
*H2 Demand authenticity has a strong moderating effect on the relationship between food neophilia and (a) food quality; (b) service quality; (c) FLEs attitude; (d) atmosphere; (e) price in the choice of an ethnic restaurant*
*H3 (a) age; (b) gender; (c) marital status; (d) financial well-being; and (e) race/ethnicity have a positive effect on the relative importance of the key dining attributes*: *food quality; service quality; FLEs attitude; atmosphere; price in the choice of an ethnic restaurant*

## Materials and methods

A self-administered online survey was undertaken for the data collection process that was divided into two batches. Batch 1 was fielded from November 2020 to January 2021, while Batch 2 was in field from July 2022 to October 2022. The survey link was disseminated randomly through online public communities on social media sites (e.g., Facebook groups for students, workers, expatriates in largest cities in Hungary). The study involved convenience samples of regular full-service restaurant customers living in Hungary with no specific inclusion and exclusion criterion in defining an eligible study participant to enrol, except for the age limits: those under age 18 were not recruited. A total of 403 responses were collected and used for the data analysis. No invalid or unusable responses were recorded.

The survey instrument comprised three sections. The first section included questions related to restaurant dining attributes where the respondents were asked to indicate their agreement with each statement using a 7 point-Likert scale anchored from ‘Strongly disagree’ to ‘Strongly agree’. From the literature review, key five dimensions of restaurant dining attributes were identified: food quality, service quality, atmosphere, frontline staff attitude and price. In the second section, the respondents were asked to rate their level of likeliness to try ethnic food of various cultures and demand authenticity on a 7 point-Likert scale with 1 being ‘Not at all likely’ and 7 being ‘Extremely likely’. At the end of the survey, the respondents were asked questions related to their demographic information.

Data were analysed using SPSS Statistics 24.0 (2016, SPSS Inc., Chicago, IL) at *p* < 0.05. Descriptive statistics were used to summarize participants’ demographic profiles and distribution of food neophilia and demand authenticity levels. In order to further understand the key dining attributes influenced by ethnic food neophilia in the choice of a full-service restaurant, General Linear Method–MANCOVA was run. In MANCOVA, the third variables called covariates are added to eliminate these variables’ effects on the relationship between independent variables (e.g., food neophilia level; demand authenticity level) and dependent variables (e.g., key dining attributes). Standardized *β* coefficients were used to compare the relative importance of each coefficient in a regression model.

## Results

Table 2 illustrates the distribution of food neophilia level as well as demand authenticity level. From the total of 403 participants, 99% (400) had dined at a full-service ethnic restaurant in the past one year, whereas 1% (3) of the participants had not dined at one. These three participants had at least one visit to an ethnic restaurant in the past two years. Among these 403 participants, both had and had not dined at a full-service ethnic restaurant in the past one year, 76% (305) had a high tendency to explore ethnic foods of different cultures, 20% (81) showed only a moderate level of food neophilia, and 4% (17) had little or no interest in enjoying ethnic food. Furthermore, a slightly more than half of the participants (55%) expressed that they seek for authenticity in ethnic restaurants, while 37% (148) demonstrated moderate preference for authenticity and 8% (33) said that authentic does not necessarily align with quality.

**Table 2:** Distribution of food neophilia and demand authenticity levels.

Variable	Category	N (%)
Food neophilia level	High	305 (76%)
	Moderate	81 (20%)
	Low	17 (4%)
	Total	403 (100%)
Demand authenticity level	High	222 (55%)
	Moderate	148 (37%)
	Low	33 (8%)
	Total	403 (100%)

Demographic profiles of the participants are summarized in Table 3. As shown in Table 3, there was a slightly larger proportion of female participants (57%) in the sample. The highest age group was between 24 and 39 (56%), followed by 18–23 (34%). The most dominant race/ethnicity group was European (51%), followed by Asian (24%). Over half of the participants (63%) were single, followed by those who were married/in a domestic partnership (35%). The majority of participants were financially secure and able to save a little for short-term goals (42%), followed by slightly over a quarter who were financially stable (29%) where they could easily make ends meet.

**Table 3:** Demographic profiles of the participants.

Variable, Category	N (count)	%	Variable, Category	N (count)	%
Gender			Marital status		
Female	230	57%	Single	254	63%
Male	173	43%	Married/in a domestic partnership	142	35%
Total	403	100%	Divorced/widowed	7	2%
Age			Total	403	100%
Gen Z (18–23)	139	34%	Financial well-being		
Gen Y (24–39)	226	56%	Fragile (I hardly make ends meet)	45	11%
Gen X (40–55)	32	8%	Stable (I can easily make ends meet)	118	29%
Baby boomer (> 56)	6	1%	Secure (I can save a little for short-term goals)	168	42%
Total	403	100%	Freedom (I have financial plans for long-term goals)	27	7%
Race/Ethnicity			Fulfil (I live the life I want without financial stress)	45	11%
African	33	8%	Total	403	100%
American	23	6%			
Asian	96	24%			
European	205	51%			
Middle eastern	34	8%			
Other	12	3%			
Total	403	100%			

### The effect of food neophilia, authenticity, and demographic characteristics

A multivariate analysis of covariance (MANCOVA) was conducted to investigate the effects of food neophilia level on the relative importance of the key dining attributes. Five key dining attributes were included as dependent variables: the importance of food quality, service quality, FLEs attitude, atmosphere, and price when choosing an ethnic restaurant. Food neophilia and demand authenticity level were measured as independent variables, while demographic characteristics (age, gender, marital status, financial well-being, and race/ethnicity) as covariates. As reported in Table 4, only some covariates were significant (marital status: *F*[5355] = 3.462, *p*<0.010; financial well-being: *F*[5355] = 3.238, *p*<0.010; race/ethnicity: *F*[5355] = 0.838, *p*<0.050). Therefore, H3a and H3b were rejected, indicating that there were no effects of age and gender on the relationship between food neophilia and the relative importance of the key dining attributes. Moreover, significant main effects of the two independent variables as well as their interaction effects on dependent variables were found at both the multivariate (food neophilia: *F*[301422] = 2.753, *p*<0.010; demand authenticity: *F*[301422] = 1.853, *p*<0.001; food neophilia x demand authenticity: *F*[1301754] = 1.614, *p*<0.001) and univariate levels (see Tables 4 and 5).

**Table 4:** Summary of MANCOVA results.

Effect	Wilks’ *λ*	df_1_	df_2_	*F*	*p*	*η*_p_^2^
AG	.991	5	355	.620	.685	.009
GN	.973	5	355	1.943	.087	.027
MR	.954	5	355	3.462	.005**	.046
FW	.956	5	355	3.238	.007**	.044
RC	.988	5	355	.838	.011*	.078
FN	.798	30	1422	2.753	.003**	.030
AT	.858	30	1422	1.853	.000***	.044
FN x AT	.573	130	1754	1.614	.000***	.105

*Note*: AG: Age; GN: Gender; MR: Marital Status; FW: Financial Well-Being; RC: Race/Ethnicity; FN: Food Neophilia; AT: Demand authenticity.

*p<0.050;

**p<0.010;

***p<0.001.

**Table 5:** A two-way ANOVA results.

	Dependent variable														
	Food quality			Service quality			FLEs attitude			Atmosphere			Price		
Source	*F*	*p*	*η*_p_^2^	*F*	*p*	*η*_p_^2^	*F*	*p*	*η*_p_^2^	*F*	*p*	*η*_p_^2^	*F*	*p*	*η*_p_^2^
MR	1.269	.261	.004	2.486	.116	.007	.013	.911	.000	.289	.591	.001	8.268	.004**	.023
FW	.262	.609	.001	2.374	.124	.007	4.847	.028*	.013	1.353	.245	.004	7.161	.008**	.020
RC	2.528	.113	.007	4.050	.046*	.022	.300	.585	.000	.523	.470	.001	.108	.742	.000
FN	3.619	.002**	.057	1.752	.108	.028	2.908	.025*	.046	8.023	.000***	.118	.765	.598	.013
AT	2.988	.000***	.048	2.486	.023*	.040	1.710	.009**	.028	1.542	.000***	.025	2.797	.011*	.045
FN x AT	2.494	.000***	.153	1.581	.037*	.103	1.737	.015*	.112	2.536	.000***	.155	1.384	.103	.091

*Note*: FW: Financial Well-Being; RC: Race/Ethnicity; FN: Food Neophilia; AT: Demand authenticity.

*p<0.050;

**p<0.010;

***p<0.001.

### The relative importance of the key dining attributes

First, concerning demographic characteristics in within subject analysis, as shown in Table 5, marital status (*F* = 8.628, *p*<0.010) and financial well-being (*F* = 7.161, *p*<0.010) affected the importance level of price. Additionally, financial well-being did also influence FLEs attitude in the model (*F* = 4.847, *p*<0.050). Thus, H3c and H3d were partially supported. Race/ethnicity showed a significant interaction with service quality (*F* = 4.050, *p*<0.050), which partly supported H3e. Second, food neophilia as a main independent variable was predicted to show significant influences on all key dining attributes. However, the results show that food neophilia level, on itself, only had significant effects on food quality (*F* = 3.619, *p*<0.010), FLEs attitude (*F* = 2.908, *p*<0.050) and atmosphere (*F* = 8.023, *p*<0.001). Therefore, H1 was partially supported. Authenticity, on the other hand, showed significant effects on all key dining attributes (food quality: *F* = 2.988, *p*<0.001; service quality: *F* = 2.486, *p*<0.050; FLEs attitude: *F* = 1.710, *p*<0.010; atmosphere: *F* = 1.542, *p*<0.001; price: *F* = 2.797, *p*<0.050). Further, in addition to finding the main effects of food neophilia, interaction effects between food neophilia and authenticity were also observed. As reported in Table 5, when interacted with authenticity, not only did food neophilia influence food quality, FLEs attitude and atmosphere, but the interaction between the two independent variables influenced service quality (*F* = 1.581, *p*<0.050), providing sufficient evidence to declare H2a, H2b, H2c, and H2e were true. This finding confirms that authenticity remains a key component in the ethnic restaurant decision-making process. The statistically significance of the main effects, thus, must be interpreted in the context of a significant two-way interaction between food neophilia and demand authenticity.

In between subject analysis, results corresponding to the interaction effects between participants with high food neophilia and a high versus a lower need for authenticity are depicted in Table 6. Among five key dining attributes that were measured, four were found to be significantly influenced by the interaction of the two predictors high food neophilia x a high need for authenticity: food quality, service quality, FLEs attitude, and atmosphere. Price, which was not found significantly affected by the interaction between food neophilia and demand authenticity in the within-subject analysis, also showed no significant relationship in the interaction between high food neophilia and a high need for authenticity. However, when high food neophilia interacted with a lower need for authenticity, the two predictors influenced only food quality and price, serving as proof that the importance of price in ethnic restaurants is more likely to vary depending on individuals’ level of food neophilia and needs for authenticity.

**Table 6:** The interaction between high food neophilia and a high vs lower need for authenticity.

	High food neophilia x High need for authenticity					High food neophilia x Lower need for authenticity				
	Mean	SD	*t*	*p*	*β*	Mean	SD	*t*	*p*	*β*
Food quality	6.13	.11	5.11	.000***	.46	5.62	.85	3.79	.000***	.36
Service quality	5.70	.25	3.60	.007**	.20	5.12	.62	1.53	.127	.08
FLEs attitude	5.74	.44	2.75	.000***	.27	5.37	.54	1.34	.179	.07
Atmosphere	5.89	.02	4.51	.000***	.41	5.01	.76	.41	.682	.02
Price	5.03	.16	1.73	.085	.10	4.97	.75	4.47	.000***	.41

*Note*: High food neophilia = T2B (Extremely likely, Likely)

High need for authenticity = T2B (Extremely likely, Likely); Lower need for authenticity = Bottom 5 boxes

*p<0.050;

**p<0.010;

***p<0.001

Moreover, food quality was the most important determining factor of overall dining experience and repeat patronage in ethnic restaurants for individuals with high food neophilia and a high need for authenticity (*β =* .46, *p*<0.001), followed by atmosphere (*β =* .41, *p*<0.001), FLEs attitude (*β =* .27, *p*<0.001), and service quality (*β =* .20, *p*<0.010).

## Discussion

The current study examined the effects of food neophilia level on the relative importance of the five key dining attributes: food quality, service quality, FLEs attitude, atmosphere, and price. In addition, the interaction effects between food neophilia and the needs for authenticity were also tested. Previous studies have observed the role of food neophobia as a determinant of food choice and preferences in highly diverse multicultural societies [4, 14, 30, 82–88]. Research on the role of food neophilia in determining the relative importance of ethnic dining-experience attributes, however, is lacking. This neglect is surprising as food neophilic individuals have higher tendencies to accept and try novel foods. Moreover, even though authenticity has been widely analyzed in the topic of ethnic food consumption [79, 89, 90], previous researchers have rarely examined the moderating role of authenticity in food neophilics’ choice of an ethnic restaurant. Therefore, findings of this study should provide interesting theoretical and managerial implications.

This research makes several academic contributions. First, in overall, this study enriches the understanding of ethnic dining experiences from the perspective of food neophilia and the needs for authenticity. Although existing research has posited that food quality and service quality are the most important determining factors of overall dining experience and repeat patronage [36, 41], this study found that food quality, atmosphere, and the friendliness of the frontline staff are the hygiene factors in ethnic restaurants. The accuracy and timeliness of the frontline staff, however, is only perceived as important when moderated by authenticity. Further, [35, 91] have identified that price fairness or value for money as an important factor in consumers’ restaurant buying decision process. However, results of this study found that food neophilia does not explain the importance of price when making a buying decision. The interaction effects between food neophilia and demand authenticity also showed a consistency that the two predictors did not affect the importance of price. Interestingly, on its own, the level of authenticity had a positive correlation with price, where the participants’ perception toward the importance of price fairness were significantly increased, especially in individuals with a lower need for authenticity.

In between-subject analysis, the results of interaction effects between participants with high food neophilia and a high need for authenticity indicate that individuals’ level of food neophilia and needs for authenticity will likely influence customers’ perception of price fairness. Based on this study’s findings, the importance of price fairness is also influenced by individuals’ marital status and financial well-being. Participants with high food neophilia and a high need for authenticity did not consider price as an important factor when choosing an ethnic restaurant. It contradicts existing research that in the decision-making process, customers considered price as one of the important factors in restaurants in general and ethnic restaurants in particular [92, 93]. This surprising finding could also be explained by the participants’ marital status and financial well-being situation that were skewed to the non-price sensitive segments, those who were single and financially secure and stable. In addition, as authentic customers are those who are eager to indulge in a culinary experience [39], they are likely to be willing to pay extra for authentic ethnic fare. Technomic’s 2018 Ethnic Food & Beverage Consumer Trend Report highlighted that 87% of US consumers have dined at an ethnic restaurant and have ordered food with ethnic flavors. One third of these consumers ordered ethnic food at least once a week and 32% allocated extra budget for an authentic ethnic fare [94].

These findings serve as an important contribution to extant research that authenticity has become a critical buying factor for customers in evaluating a product or service [95]. Moreover, [96] argued that authenticity in ethnic restaurants is a fundamental value that appeals customers. [9] found that a lack of authenticity is one of the triggers that causes hospitality and service businesses are failed.

In regard to food authenticity, this study’s results also promote previous empirical research in which customers were found to be more interested about the food authenticity than atmosphere in ethnic restaurants [7, 8]. Undoubtedly, authentic food is a critical element of a restaurant dining experience, particularly in the context of ethnic restaurant. According to [51], authentic food customers enjoy experiencing different cultures and are interested in trying different culinary traditions. These customers also prefer to choose an ethnic restaurant where the natives dine and usually have a good cultural knowledge which makes them able to identify the real from the fake apart [76]. That is the reason why, to generate positive behavioral intentions in ethnic restaurants, both authentic food and authentic atmosphere cannot be seen in isolation from one another. As [38] noted in their empirical research that authentic atmosphere significantly triggers positive emotions and may lead to future purchase intentions, therefore, it is understandable that those participants with a high need for authenticity perceived food quality and atmosphere to be superior to other key dining attributes.

Furthermore, according to the participants, authentic atmosphere was ranked as one of the most important factors in ethnic restaurants. These findings empirically support existing studies that ethnic theme influences customers’ perception of food authenticity, acceptability, and how they select food in ethnic restaurants [63]. [97] found that atmosphere and food appearance were the most important factors and were inseparable from each other in Indian restaurants in America. [98] elicited that atmosphere is a fundamental construct in dining experience that can lead to positive emotion, satisfaction, and re-patronage intentions. It is also supported by [46], that the atmosphere of the place in some cases to be more powerful than the product or service itself. [99] outlined that atmospheric also have significant effects on customers’ food selection behavior, food perception, and consumption patterns.

The least important attribute but also significantly affected by the two predictors are FLEs attitude and service quality. [100] indicated that frontline staff’s working time are spent mainly on service encounters. They highly contribute to the professional aspect of experience [101] as well as create the enjoyable service interactions [102]. [103] reported that frontline staff can compensate the core service failure by creating an enjoyable customer-employee interaction. According to [104], the determinant of service quality is directly related to the frontline staff. However, frontline staff attitude (e.g., friendliness, politeness, willingness to help) is frequently combined with prompt service, accuracy, and consistency as a single attribute: service quality, and only few researchers conform on its dimensionality. This study examined these factors as two separate ones because they serve two different purposes and exert differential effects on customer satisfaction. First, friendliness, politeness, and willingness to help–simultaneously, serve more as affective attributes, while service quality dimensions, for example prompt service, accuracy, and consistency serve as functional attributes. In both Multivariate and Univariate analyses, FLEs attitude was found significantly influenced by food neophilia in itself and its interaction with demand authenticity. Moreover, in the interaction between high food neophilia and a high need for authenticity, FLEs attitude was also among those significant factors influencing ethnic restaurant selection. That is because, as an affective and social component of the customer experience [105], FLEs attitude is highly likely to generate either positive or negative emotions. Positive employee attitude generates customer positive emotional experiences [106] and enhances customer-employee interpersonal relationship [107]. Furthermore, [108] argued that FLEs attitude is a crucial determining factor of relationship marketing, which is a strategy of Customer Relationship Management (CRM) as FLEs attitude conveys an affective social interaction between the customer and the employee. In ethnic restaurants, customers who dine in would like to develop deeper connections with a certain culture, not only through tangible factors, but also non-physical factors such as frontline employees. Based on this study’s findings as well as previous empirical research, it is wise to conclude that FLEs attitude is a hygiene factor in the hospitality and restaurant industry. As [109] highlighted in their study, “A truly authentic pub is not just about the design, cultural artifacts, or music, it is about the people who work and patronize it.”

Service quality (e.g., prompt service, accuracy, consistency), on the other hand, did not significantly influenced by food neophilia in solitude, but it seemed, however, to be affected by authenticity, also its interaction with food neophilia and one demographic characteristic ‘race/ethnicity’ as covariate. However, the interaction between a lower need for authenticity and high food neophilia did not show any significant correlation. [110] explained that customer evaluation of service encounter is shaped by culture. Culture also has direct influences on norms, and how service is perceived corresponds to different cultural norms by which these norms guide the behavior [111]. Thus, service quality might be perceived differently depending on the individuals and the cultural contexts. In a study by [112], customers who were asked to evaluate between Finnish and Nepalese staff at a Nepalese restaurant in Finland expressed that Finnish staff take the orders accurately and have strong professional skills, but fast paced and straightforward in their interactions with customers as well as relatively less friendly compared to Nepalese staff. On the contrary, many customers prefer Nepalese staff for their “relaxing and friendly way of serving”. In other cases, Japanese customers prefer roles and competence rather than individuals, thereby, distant customer-employee relationship is more embraced [111]. Not one restaurant business wants their staff to deliver service with long wait times as the wait times may increase both monetary and psychological costs for customers [58], yet a fast-paced service can ruin customers’ pleasure in their dining experience [113]. In other words, restaurant service is segment-specific; restaurants are classified into different types of services [114]. In ethnic restaurants, customers want to indulge in a totally different culture and seek the cultural authenticity [79], therefore the prompt service, accuracy, and consistency alone are not favored.

## Conclusion

This study allows a better understanding of food neophilics segment and their attitude toward ethnic restaurants. A few questions invigorated this study, what are the most important attributes for these customers when choosing an ethnic restaurant? How this market segment makes a purchase decision? In which the relationship between the predictors and the response variables was also understood by adding a moderation role of demand authenticity and demographic characteristics as covariates in statistical modelling. The interaction effects between food neophilia, the needs for authenticity, and demographic characteristics in influencing five key dining attributes are summarized in Table 7.

**Table 7:** Summary of hypotheses tests.

Hypotheses	Results	Remark
H1a: food neophilia enhances the importance of food quality	Supported	Food neophilia significantly increased participants’ perception of the importance of food quality
H1b: food neophilia enhances the importance of service quality	Unsupported	No significant impact found
H1c: food neophilia enhances the importance of FLEs attitude	Supported	Food neophilia significantly increased participants’ perception of the importance of FLEs attitude
H1d: food neophilia enhances the importance of atmosphere	Supported	Food neophilia significantly increased participants’ perception of the importance of atmosphere in ethnic restaurants
H1e: food neophilia enhances the importance of price	Unsupported	No significant impact found
H2a: demand authenticity moderates the effect of food neophilia on food quality	Supported	The interaction effect between food neophilia and the needs for authenticity significantly increased the perception of food quality’s importance in ethnic dining experience
H2b: demand authenticity moderates the effect of food neophilia on service quality	Supported	The interaction effect between food neophilia and the needs for authenticity significantly increased the perception of service quality’s importance in ethnic dining experience
H2c: demand authenticity moderates the effect of food neophilia on FLEs attitude	Supported	The interaction effect between food neophilia and the needs for authenticity significantly increased the importance of FLEs attitude in the choice of an ethnic restaurant
H2d: demand authenticity moderates the effect of food neophilia on atmosphere	Supported	The interaction effect between food neophilia and the needs for authenticity significantly increased the importance of atmosphere in the choice of an ethnic restaurant
H2e: demand authenticity moderates the effect of food neophilia on price	Unsupported	No significant impact found
H3a: age affects the effect of food neophilia on key dining attributes	Unsupported	No significant impact found
H3b: gender affects the effect of food neophilia on key dining attributes	Unsupported	No significant impact found
H3c: marital status affects the effect of food neophilia on key dining attributes	Partially supported	Marital status significantly contributed to influencing the importance of price in ethnic restaurants
H3d: financial well-being affects the effect of food neophilia on key dining attributes	Partially supported	Financial well-being significantly contributed to influencing the importance of FLEs attitude and price in ethnic restaurants
H3e: race/ethnicity affects the effect of food neophilia on key dining attributes	Partially supported	Race/ethnicity significantly contributed to influencing the importance of service quality in ethnic restaurants

To keep up with fierce competition in the market, offering authentic quality of food; authentic theme that reflects cultural elements; a friendly, relaxing, but also prompt service environment is critical in providing ethnic restaurants with a competitive advantage, particularly if the market segment includes food neophilic customers with a high demand for authenticity. For these segments, the salience of price in buying decisions is low as customers are willing to pay extra and less likely to fixate on price for a good dining experience. Considering that these types of customers want to indulge in different cultural and culinary traditions, a casual fast-paced service restaurant may not be favored. Conversely, price sensitivity will be higher in a more intense price-based competition, for example a market where customers do not crave for authenticity. In other words, if the core customer base does not include customers with a high demand for authenticity, restaurant managers should factor in ‘price’ in the customer buying decision. Hence, an upscale ethnic restaurant may not be suitable for these customers.

Ethnic restaurant customers who like to explore a variety of foods and value authenticity offered in ethnic restaurants seem to also appreciate pleasant exchanges with the frontline staff; therefore, accurate but friendly and relaxing service encounters will be more favorable than providing a meticulous yet straightforward and fast-paced service, especially in a more casual establishment. By developing recruitment; training; and performance monitoring standards that emphasize friendliness, empathy, and willingness to help, restaurant managers could equip their frontline staff with Customer Relationship Management (CRM) knowledge to optimize overall service quality that leads to customer satisfaction and re-patronage intentions.

Despite the contributions of the findings, a few limitations in this study should be noted. First, as this study focuses solely on ethnic restaurants, the results may not be directly applicable to non-ethnic restaurants. Additionally, this study is limited to the discussion of food neophilia, thus, the results may only be generalizable to this market segment, given restaurant customers may have different expectations and consider different factors in the restaurant selection. Moreover, the salience of restaurant dining experience factors may also vary than those that were measured in this study. Second, the research sample was drawn in Hungary, hence, outcomes may not transfer automatically from one service context to another.

Future researchers could undertake a comparative study between food neophilic individuals and food neophobic individuals in the choice of a full-service restaurant or other types of restaurants (e.g., fast food, casual dining, fine-dining, family style) which also incorporates other boundary conditions, such as psychographic characteristics. To broaden the study scope, future research could also identify the salience of affective-specific attributes versus functional-specific attributes on the subject of restaurant dining experience. In addition, an empirical investigation towards the moderating role of a high demand for healthy foods is also desirable for future research as increasing awareness of healthy lifestyle is anticipated to drive the demand. Additional service contexts and attributes could provide a more thorough justification and contribution to the restaurant selection and dining experience constructs.

## Supporting information

S1 Appendix Indicators. (DOCX)
