# Supplementary material for: Food neophilics’ choice of an ethnic restaurant: The moderating role of authenticity
Source: PLoS One. 2023 May 18;18(5):e0281453. doi: 10.1371/journal.pone.0281453 (PMC10194881; doi:10.1371/journal.pone.0281453)
Supplement: S1 Appendix — (DOCX) [file pone.0281453.s001.docx]

**S1 Appendix. Indicators.**

| **Food neophilia** (*M = 5.97; SD = 1.37; CV = 22.90; Skewness = -1.79*) | |
| --- | --- |
| FN1 | I like to try new ethnic restaurants |
| FN2 | I am constantly sampling new and different foods |
| FN3 | I like to experience food of various cultures and traditions |
| FN4 | I am not afraid to eat things I have never had before |
| FN5 | I will eat almost anything |
| **Demand authenticity** (*M = 5.25; SD = 1.54; CV = 29.28; Skewness = -0.96*) | |
| AT1 | I like to dine in at an ethnic restaurant that embodies the essence of a specific region |
| AT2 | I like to dine in at an ethnic restaurant which serves food that makes me feel like I'm dining in the region where it originated from |
| AT3 | I like to dine in at an ethnic restaurant which uses cooking techniques unique to a specific region |
| AT4 | I would likely choose an ethnic restaurant that appears to connect with what I know about the region |
| AT5 | I would likely choose an ethnic restaurant which serves dishes that have flavours traditional to its region |
| **Food quality** (*M = 6.00; SD = 1.43; CV = 23.87; Skewness = -1.77*) | |
| FQ1 | Appropriate food portion |
| FQ2 | Food taste, temperature and presentation |
| FQ3 | Variety of menu |
| **Service quality** (*M = 5.56; SD = 1.40; CV = 25.20; Skewness = -1.18*) | |
| SQ1 | The food is served exactly as ordered and when something is wrong, it is corrected properly |
| SQ2 | Staff competency, reliability and consistency |
| SQ3 | Timeliness of the service |
| **FLEs attitude** (*M = 5.88; SD = 1.53; CV = 26.05; Skewness = -1.72*) | |
| FLE1 | Staff is willing to help, sympathetic, calm |
| FLE2 | Staff friendliness |
| FLE3 | Staff hygiene, clean uniforms and appearance |
| **Atmosphere** (*M = 5.67; SD = 1.37; CV = 24.16; Skewness = -1.54*) | |
| ATM1 | Cleanliness of the dining area and toilet facilities |
| ATM2 | Comfort of the dining area (adequate dining tables, chairs, space) |
| ATM3 | Pleasant décor (adequate lighting, aroma/scents, music quality and volume) |
| **Price** (*M = 5.02; SD = 1.64; CV = 32.63; Skewness = -0.79*) | |
| PR1 | Value for money |
| PR2 | Offer good deals |
| PR3 | No hidden costs (service charges, taxes, packing fees) |

*NOTE. All variables are measured on 7-point Likert-type scales (1 = strongly disagree, 7 = strongly agree, with the exception of food neophilia and demand authenticity: 1 = not at all likely, 7 = extremely likely)*
